# Evaluation of Penicillin Allergy Prevalence and Antibiotic Prescribing Patterns for Patients within the Emergency Department

**DOI:** 10.1017/ash.2021.70

**Published:** 2021-07-29

**Authors:** Ashlyn Norris, Lindsay Daniels, Nikolaos Mavrogiorgos, Kalynn Northam, Mildred Kwan, Gary Burke, Renae Boerneke

## Abstract

As the point of entry into healthcare for many patients, the emergency department (ED) is an ideal setting in which to assess penicillin (PCN) allergies. An estimated 10% of the United States population has a reported PCN allergy; however, few studies have evaluated the prevalence and impact of PCN allergies on antibiotic selection within the ED. Patients with a documented PCN allergy are more likely to be exposed to costly alternative broad-spectrum antibiotics that have higher rates of adverse events, including *C. difficile* infections. We sought to determine the prevalence of PCN allergies within the UNC Medical Center ED. Key secondary outcomes included the percentage of patients with a documented PCN allergy who (1) received alternative antibiotics (carbapenems, aztreonam, fluoroquinolones, clindamycin, vancomycin), (2) received β-lactam antibiotics and experienced an allergic reaction during their ED visit, and/or (3) had received a β-lactam antibiotic during a past hospitalization or ED visit without their chart being appropriately updated. A retrospective evaluation included patients aged >18 years with a documented PCN allergy who were discharged from the ED between January 1, 2017, and December 31, 2019. Over the study period, there were 14,635 patient encounters with a documented PCN allergy that comprised 8,573 unique patients. The prevalence of PCN allergies was 14.3% for all ED encounters. PCN allergy–labeled patients received alternative antibiotics in 59.4% of ED encounters in which antibiotics were prescribed. Of the 454 β-lactam antibiotics (62 penicillins, 380 cephalosporins, 12 carbapenems) administered to PCN allergy-labeled patients within the ED, there were zero allergic reactions. Also, 18.6% of PCN allergy-labeled patients had received and tolerated a β-lactam antibiotic during prior hospitalizations or ED visits (1.7% penicillins, 14.4% cephalosporins, 2.6% carbapenems) without appropriate updated documentation to reflect β-lactam antibiotic tolerance. These findings confirm the utilization of non–β-lactam antibiotics in PCN allergy-labeled patients, highlighting the importance of accurate and updated allergy documentation in the electronic medical record. These findings also demonstrate the need for improved allergy documentation and protocols to proactively assess penicillin allergy labels while in the ED.

**Funding:** No

**Disclosures:** None

